# Exosomes: The Link between GPCR Activation and Metastatic Potential?

**DOI:** 10.3389/fgene.2016.00056

**Published:** 2016-04-08

**Authors:** Allison L. Isola, Suzie Chen

**Affiliations:** ^1^Susan Lehman Cullman Laboratory for Cancer Research, Department of Chemical Biology, Ernest Mario School of Pharmacy, Rutgers the State UniversityPiscataway, NJ, USA; ^2^Joint Graduate Program in Toxicology, Environmental and Occupational Health Sciences Institute, Rutgers the State UniversityPiscataway, NJ, USA; ^3^Rutgers Cancer Institute of New JerseyNew Brunswick, NJ, USA

**Keywords:** GPCR, exosome, pre-metastatic niche, cancer, mGluR

## Abstract

The activation of G-Protein Coupled Receptors (GPCRs) by their respective ligands initiates a cascade of multiple signaling processes within the cell, regulating growth, metabolism and other essential cellular functions. Dysregulation and aberrant expression of these GPCRs and their subsequent signaling cascades are associated with many different types of pathologies, including cancer. The main life threatening complication in patients diagnosed with cancer is the dissemination of cells from the primary tumor to distant vital organs within the body, metastasis. Communication between the primary tumor, immune system, and the site of future metastasis are some of the key events in the early stages of metastasis. It has been postulated that the communication is mediated by nanovesicles that, under non-pathological conditions, are released by normal cells to relay signals to other cells in the body. These nanovesicles are called exosomes, and are utilized by the tumor cell to influence changes within the recipient cell, such as bone marrow progenitor cells, and cells within the site of future metastatic growth, in order to prepare the site for colonization. Tumor cells have been shown to release an increased number of exosomes when compared to their normal cell counterpart. Exosome production and release are regulated by proteins involved in localization, degradation and size of the multivesicular body, whose function may be altered within cancer cells, resulting in the release of an increased number of these vesicles. This review investigates the possibility of GPCR signaling cascades acting as the upstream activator of proteins involved in exosome production and release, linking a commonly targeted trans-membrane protein class with cellular communication utilized by tumor cells in early stages of metastasis.

## Introduction

Increasing evidence links the aberrant protein expression of G-protein coupled receptors with numerous pathologies, including cancer. Exosomes are membrane-bound nanovesicles that have been implicated as an important component in preparing distal organs for tumor cell metastasis. This review intends to explore and speculate about G-protein coupled receptors and their links to cancer, exosomes, and the involvement in cancer metastasis.

## G-protein coupled receptors

Guanine nucleotide binding-protein coupled receptors (GPCRs) make up the largest family of proteins found within the mammalian genome (Lander et al., 2001; Venter et al., 2001). The GPCR superfamily contains over 800 different seven trans-membrane receptors. Two requirements must be met in order to be classified as a GPCR; the first is that the receptor contains seven stretches of about 30 highly hydrophobic residues that represent trans-membrane locations, which provide the protein with both intracellular domains and an extracellular domain that has the ability to interact with its ligand. The second requirement that defines a GPCR is interaction with guanine nucleotide binding proteins (G-proteins). GPCR classification within the superfamily is based on how the ligand binds to the receptor, physiological, and structural features of the receptor, as well as phylogenetics. The most frequently used classification system is A, B, C, D, E, and F (Attwood and Findlay, 1994; Kolakowski, 1994) which represent GPCRs from all living beings from humans to bacteria. The majority of human GPCRs are separated into 5 different families; glutamate, rhodopsin, adhesion, frizzled/taste2, and secretin (GRAFS nomenclature; Fredriksson et al., 2003; Lagerstrom and Schioth, 2008).

The natural ligands for GPCRs vary from ions, proteins, lipids, hormones, neurotransmitters, amines, nucleotides, odorant molecules to photons. GPCRs are associated with heterotrimeric G-protein subunits consisting of G_α_, G_β_, and G_γ_, that function as dimers at the intracellular domain of the GPCR. Once the ligand binds to the receptor, it causes a conformational change, activating the receptor and initiating an intracellular cascade. The inactive form of the receptor is bound to guanine diphosphate (GDP), and this conformational change results in the exchange of GDP with guanine triphosphate (GTP) of the associated G-protein within the intracellular domain of the GPCR. This phosphate exchange alters the affinity of the G-protein with the GPCR and results in the dissociation of that G-protein (Hamm, 1998; Bunemann et al., 2003), GPCRs can then interact with a multitude of different targets including ion channels, tyrosine kinases, adenylyl cyclases, phosphodiesterases, and others (Lee et al., 2008; Lappano and Maggiolini, 2011). Disruption in the function of GPCRs are known to be responsible for many prevalent human diseases such as nephrogenic diabetes insipidus (Spiegel, 1996a), cardiovascular disease (Hata and Koch, 2003), endocrine diseases (Spiegel, 1996b; Lee et al., 2008; Lappano and Maggiolini, 2011), and others.

The GPCRs whose natural ligands are neurotransmitters, specifically glutamate, are classified under class C receptors (Bjarnadottir et al., 2005), and are broken down into metabotropic glutamate receptors (mGluR), GABA receptors, calcium sensing receptors, taste receptors, and some orphan receptors (Wu et al., 2014). The remainder of this review focuses on the metabotropic glutamate receptors (mGluRs) particularly mGluR1. The mGluRs can be broken down into groups I through III, based on their sequence homology, pharmacologic responses, and intracellular second messengers. Group I consists of mGluR1 and mGluR5, group II contains mGluR2 and mGluR3, and group III contains mGluR4, mGluR6, mGluR7, and mGluR8 (Nakanishi, 1992). Binding of the ligand, glutamate, to group I mGluRs resulted in exchange of GTP for GDP on G_α_. Specifically, groups II and III mGluRs are coupled to G_α*i/o*_. Group I mGluR activation results in the stimulation of phospholipase C β (PLCβ) which cleaves phosphatidylinositol 4,5-bisphosphate (PIP_2_) into two second messengers: inositol triphosphate (IP_3_), which are released into the cytoplasm, and diacylgycerol (DAG), which remains associated with the plasma membrane. Discharged IP_3_ initiates the activation of protein kinase C (PKC), which is involved in phosphorylation of various proteins to participate in numerous cellular functions. The hydrolysis to the second messenger IP_3_ results in the mobilization of calcium from the endoplasmic reticulum, and the subsequent activation of various calcium dependent kinases (Marin and Chen, 2004). The group II and III mGluRs associated G_α*i/o*_, once activated, prevent the formation of cAMP by inhibiting adenylyl cyclase activity.

## GPCRs and cancer

The first report identifying a GPCR as an oncogene was in 1986 by Wigler and co-workers when they demonstrated the transforming activity of a rat protein, MAS (Young et al., 1986). Unlike most oncogenes identified at that time, MAS did not have activating mutations. Subsequent studies showed that the ability of GPCRs to possess oncogenic potential is by either aberrant protein expression or the excessive local production of ligands by tumor cells themselves (autocrine) or stromal counterparts (paracrine) and increasing the available ligand and subsequent receptor activation (Young et al., 1986). Mutations have also been detected in GPCRs, including a gain of function mutation causing amino-acid changes in G-proteins where GTP is bound. These mutations can initiate signaling cascades independent of GPCR activation (Van Raamsdonk et al., 2004).

Our laboratory was the first to suggest the role of dysregulated glutamatergic signaling in melanoma pathogenesis, which was subsequently confirmed by other investigators. It was discovered that a gain-of-function of the murine form of a neuronal receptor, metabotropic glutamate receptor 1 (gene: *GRM1*, protein: mGluR1), when ectopically expressed in melanocytes, was sufficient to induce *in vitro* melanocytic transformation and spontaneous malignant melanoma development *in vivo* in a transgenic mouse model, TG-3 (Pollock et al., 2003; Ohtani et al., 2008; Shin et al., 2008). Subsequent investigation revealed mGluR1 expression was also detected in 80% of human melanoma cell lines and 65% of human melanoma biopsy samples at levels of protein and mRNA (Pollock et al., 2003). Earlier studies showed the aberrant protein expression of GPCRs and the availability of abundant ligand in the surrounding environment are involved in cell transformation (Julius et al., 1989). We assessed levels of extra-cellular glutamate in several melanoma cell lines. We found elevated glutamate levels only in mGluR1-expressing melanoma cells (Namkoong et al., 2007). We also demonstrated stimulation of mGluR1 by its ligand, glutamate as well as other agonists, led to formation of two second messengers, DAG and IP_3_, as described in the central nervous system (CNS; Hermans and Challiss, 2001). DAG remains bound to the cell membrane, and activates PKC (Newton, 2001). PKC then activates the MAPK signaling cascade responsible for cell proliferation and inhibits apoptosis (Marin and Chen, 2004). PKC also activates the PI3K/AKT pathway (Spiegel, 1996a; Lappano and Maggiolini, 2011), which is involved in tumor cell survival, epithelial-mesenchymal transition and angiogenesis (Marin et al., 2006; Stepulak et al., 2009). Unlike many mouse models of cancer, TG-3 displays metastasis to several distal organs as the disease progresses (Zhu et al., 1998). Consequently, activation of ectopically expressed GRM1 initiates signaling cascades important for melanoma pathogenesis, which could include activation of the exosomal production pathway, paving the way for metastasis. In addition to mGluR1, other mGluRs have been implicated in numerous cancers. Table 1 summarizes various cancers associated with mGluR misregulation.

**Table 1:** **Metabotropic glutamate receptors (mGluRs) and associated malignancies**.

**Group**	**mGluR**	**Cancer**	**References**
I	mGluR1	Malignant Melanoma	Pollock et al., 2003; Marin and Chen, 2004; Ohtani et al., 2008
		Breast Cancer	Shah et al., 2012; Speyer et al., 2012; Teh et al., 2015
		Lung	Kan et al., 2010
		Ovary	Cancer Genome Atlas Research, 2008
		Large Intestine	Sjoblom et al., 2006; Wood et al., 2007; Cancer Genome Atlas Research, 2008
		Upper Aerodigestive Tract	Durinck et al., 2011; Stransky et al., 2011
		Astrocytoma	Parsons et al., 2008
		Glioma	Brocke et al., 2010
		Medulloblastoma	Brocke et al., 2010
	mGluR5	Malignant Melanoma	Frati et al., 2000; Choi et al., 2011
		Prostate	Pissimissis et al., 2009
		Oral Squamous Cell Carcinoma	Park et al., 2007
		Osteosarcoma	Kalariti et al., 2007
		Glioma	Brocke et al., 2010
		Medulloblastoma	Brocke et al., 2010
II	mGluR2	Glioma	D'Onofrio et al., 2003; Arcella et al., 2005
		Prostate	Pissimissis et al., 2009
	mGluR3	Glioma	D'Onofrio et al., 2003; Arcella et al., 2005
		Malignant Melanoma	Prickett and Samuels, 2012
III	mGluR4	Colorectal Carcinoma	Chang et al., 2005
		Glioma	Brocke et al., 2010
		Malignant Melanoma	Chang et al., 2005
		Squamous Cell Carcinoma	Chang et al., 2005
		Medulloblastoma	Iacovelli et al., 2006
	mGluR6	Glioma	Brocke et al., 2010
		Medulloblastoma	Brocke et al., 2010
	mGluR7	N/A	
	mGluR8	Malignant Melanoma	Choi et al., 2011; Prickett and Samuels, 2012

*Adapted from (Prickett and Samuels, 2012; Teh and Chen, 2012; Esseltine et al., 2013)*.

## Exosomes

Exosomes are small membrane-bound nanovesicles with the characteristic size of 30–120 nm in diameter, derived from endosomal origins, generated constitutively, and released by various cell types and more frequently by tumor cells (Thery et al., 2002). Exosomes can be found in the blood (Taylor and Gercel-Taylor, 2008), urine (Pisitkun et al., 2004), saliva (Gonzalez-Begne et al., 2009), plasma (Caby et al., 2005), breast milk (Admyre et al., 2007) as well as other bodily fluids (Andre et al., 2002; Gatti et al., 2005; Keller et al., 2007; da Silveira et al., 2012). Exosomes are actively secreted from cells by an exocytosis pathway used for receptor removal and crosstalk between cells (Stoorvogel et al., 2002; Thery et al., 2002; Valenti et al., 2007). Exosomes are shed from the surface of healthy cells, and take with them membrane proteins and cytoplasm contents of the cells they are released from including miRNAs, mRNAs, siRNAs, and proteins (Thery et al., 2002). Studies of exosomes from various cell types show several common proteins contained in all exosomes (Raposo et al., 1996; Escola et al., 1998; Thery et al., 1999, 2001; van Niel et al., 2001).

## Composition of exosomes

Exosomes contain a unique composition of proteins and nucleic acids that can vary depending on the cell type they originated from. Studies of exosomes from immature dendritic cells (DCs; Thery et al., 1999, 2001), B lymphocytes (Raposo et al., 1996; Escola et al., 1998), intestinal epithelial cells (van Niel et al., 2001), and other cell types show that there are common, as well as cell-type specific proteins residing within exosomes. Cell-type specific proteins within exosomes include Major Histocompatibility Complex (MHC) class I and II proteins, which have been detected in B lymphocyte, DCs, mast cells and intestinal epithelial cell exosomes. Von Willebrand factor (Heijnen et al., 1999), perforin and granzymes (Peters et al., 1991) were found in platelet and cytotoxic T cell exosomes, respectively. The proteins that were found to be consistent across exosome types include chaperones (Hsc73 and Hsc90), subunits of trimeric G proteins, Tsg101, cytoskeletal proteins and tetraspanins such as CD9, CD63, CD81, and CD82 (Thery et al., 1999, 2001; van Niel et al., 2001). Kahlert et al. identified double stranded genomic DNA present within exosomes (Kahlert et al., 2014).

## Formation of exosomes

One of the defining characteristics of exosomes is the endocytic origin, which sets it apart from other cellular vesicles such as apoptotic bodies that are budded off of the plasma membrane. The initial step in the formation of exosomes is endocytosis. Invagination of the plasma membrane is initiated by the deformation of the lipid bilayer, which can be influenced extrinsically or by internal membrane structural modification. Specific membrane manipulating proteins interact with and bend the membrane surface to initiate tubulation. Membranes that are tubulated experience an external force, which causes the inward curvature, or invagination of the membrane (Lipowsky, 2013). The proteins involved in this process include endocytosis proteins such as epsin (Ford et al., 2002), N-BAR proteins, such as amphiphysin (Takei et al., 1999; Peter et al., 2004) and endophilin, (Farsad et al., 2001) or F-BAR proteins, such as syndapins (Wang et al., 2009) and its associated protein, dynamin. Dynamin is a GTPase that connects with both actin and F-BAR to successfully form and cut membrane tubules to create a successful invagination of the membrane. (Reviewed by Lipowsky, 2013). Once the invaginated membrane forms and becomes severed from the plasma membrane, it is released into the cytosol of the cell as an endosome.

The Endosomal Sorting Complex Required for Transport (ESCRT) functions on the newly formed endosome to initiate the internal budding of the multivesicular bodies (MVB) membrane to form smaller intraluminal vesicles within the MVB, these vesicles are exosomes. Ceramide, a sphingolipid, was found to trigger budding of exosome vesicles into the multivesicular body (Trajkovic et al., 2008). ESCRT is made up of four different complexes (ESCRT-0, -I, -II, and -III) and associated accessory proteins. The primary function of the ESCRT proteins is to constrict the membrane, create budding within the endosome and cause severing of the budded vesicle neck to separate the vesicle from the MVB membrane. The precise mechanism of the severing is unknown. (Hurley and Hanson, 2010; Peel et al., 2011; Henne et al., 2013; McCullough et al., 2013). The proteins in the ESCRT pathway are broken up into four different complexes: ESCRT-0, -I, -II, and -III. ESCRT-0 is involved in collecting ubiquitinated proteins on the membrane of the endosome. ESCRT-I and -II initiate the inward budding of the endosomal membrane and ESCRT-III severs the budding membrane from the endosome, creating a separate smaller vesicle within the endosome; an exosome (Reviewed by Hurley and Odorizzi, 2012). ESCRTIII is recruited for scission by ALIX. Syndecans are proteins involved in sulfate-presentation on the membrane surface, and are found on exosomes. These proteins are sorted into exosomes by an adapter protein, syntenin, which binds to ALIX, recruiting ESCRTIII to finalize the formation of the exosome (Baietti et al., 2012; Hurley and Odorizzi, 2012).

The specificity of cargo sorting into these exosome vesicles is still unclear. However, it has been shown that ubiquitination serves as a signal for sorting cargo into the vesicles formed within the MVB. Additionally, evidence has shown that ESCRT-I recognizes ubiquitinated cargo, suggesting that this protein and its associated protein, Vps23, initiate MVB sorting by binding cargo and directing it to MVB for loading in a ubiquitin-binding manner (Katzmann et al., 2001).

## Exosome release

Once the MVB is formed and contains exosomes within its membrane, it has one of two fates; targeted degradation by the lysosome or plasma membrane fusion resulting in exosome release.

If the MVB is targeted for lysosomal degradation, it fuses with the lysosome and results in the release of the internal exosomes and the macromolecules contained within them, into the lumen of the lysosome. These components are then exposed to the hydrolytic enzymes within the lumen of the lysosome and are degraded (Futter et al., 1996).

Alternatively, the MVB will travel to the plasma membrane. In this case, a GTPase, RAL-1, has recently been identified to mediate the fusion of the MVB membrane with the plasma membrane of the cell to allow the release of the exosomes into the extracellular space. Syx-5 is a t-SNARE that is recruited by RAL-1 to the plasma membrane to stimulate MVB fusion. Hyenne et al., showed that without Syx-5, the MVB is unable to fuse with the plasma membrane (Hyenne et al., 2015). Ostrowski et al., identified Rab27a, Rab27b, and their effectors (SYTL4 and Slac2b, respectively) to be involved in the exosomal pathway in HeLa cells (Ostrowski et al., 2010). Specifically, Rab27a was shown to be involved in the size of the MVE, while Rab27b regulated localization of the MVB to the plasma membrane. Another Rab-GTPase, Rab35, was identified as a regulator in the docking or tethering of the MVB to the plasma membrane (Hsu et al., 2010). In addition to enzymatic involvement of exosome regulation, intracellular levels of Ca^2+^ have been shown to be proportional to exosome release (Savina et al., 2003), in addition, low pH within the microenvironment influences the release of exosomes as well as the uptake (Parolini et al., 2009).

In cancer, oncogenes have been shown to play a role in exosome secretion, including a p53-regulated pathway, TSAP6, both *in-vitro* (Yu et al., 2006) and *in-vivo* using a TSAP/Steap3-null mouse (Lespagnol et al., 2008). As tumors become more aggressive, the expression and activation of the enzyme heparanase becomes upregulated. The activation of heparanase increases the release of exosomes, as well as the cargo levels found within the exosomes (Thompson et al., 2013).

## Exosome uptake

Once the exosomes are released from the plasma membrane, they have the ability to travel to distant sites of the body, and/or interact with the cells in the surrounding microenvironment. Exosomes involved in intracellular communication contain phosphatidylserine on their outer membrane and interacts with T-cell immunoglobulin and mucin-domain-containing molecule 1 (Tim1), a transmembrane protein present on recipient cells (Thery et al., 2002). This interaction initiates the engulfment of exosomes by the recipient cell (Miyanishi et al., 2007). In ovarian cancer cells, exosome uptake was shown by clathrin-dependent endocytosis. Both proteins and specific glycoproteins present on exosomes and the cell surface were shown to be important for exosome uptake (Escrevente et al., 2011). The transfer of major histocompatibility complex (MHC)-peptide complexes between dendritic cells was shown to be dependent on the presence of intercellular adhesion molecule 1 (ICAM-1) on exosomes. Exosomes from immature dendritic cells (DCs) were unable to transfer MHC to other DCs, however, exosomes from mature DCs contained ICAM-1 on the surface of the exosomes, and resulted in transfer of MHC from the exosomes (Segura et al., 2005). Additionally, heparin sulfate proteoglycans (HSPGs) have been shown to act as receptors of tumor derived exosomes (Christianson et al., 2013). Parolini et al., were the first to show that endocytosis is not the sole route of exosome uptake. Under certain conditions, exosomes will undergo lipid-dependent membrane fusion with the recipient cell independent of energy-dependent exocytosis and protein-protein interaction (Parolini et al., 2009).

Once the exosomes enter the recipient cell, the cargo has the potential to interact and alter the physiology of the cell. Exosomes are also known to modulate gene expression as Valadi and colleagues demonstrated that RNAs in mast cell exosomes could be delivered to human and mouse mast cells leading to new protein production in recipient cells (Valadi et al., 2007).

## Exosomes in cancer

Circulating tumor cells (CTCs) are potential biomarkers for cancer; however, depending on the stage of cancer, there can be as few as 1-10 CTCs per mL of blood. Exosomes, however, are found in abundance within the blood, typically, 1 × 10^12^ exosomes per mL of blood, making them a non-invasive and ideal screening method for diagnostics, cancer progression and targeted therapy (Hyun et al., 2015). Fujita et al., suggested that exosomes have the potential to be used as biomarkers for asthma (Fujita et al., 2014). In addition to a minimally invasive biomarker, there have been efforts in using exosomes to develop a new method of drug delivery to target drug-resistant cancer. Exosome-encapsulated Paclitaxel (exoPTX) increases the cytotoxic effects on prostate cancer cells when compared to drug alone, and holds significant potential for the delivery of various chemotherapeutics to treat cancers that have became resistant to the regimen (Saari et al., 2015). In addition to drug delivery, dendritic cell-derived exosomes are being explored for their potential in cancer immunotherapy (Viaud et al., 2010). Increased exosome plasma levels are observed only in patients with advanced stage diseases (Logozzi et al., 2009; Peinado et al., 2012). Recently, an assay was developed to detect a proteoglycan molecule, glypican-1 (GPC1) found on extracellular vesicles in patients with late-stage pancreatic cancer with 100% confidence. This method is more reliable than a more common assay looking for a tumor antigen in whole blood (Thery, 2015).

Along with the potential in using these vesicles to diagnose and treat cancers, tumor exosomes have been shown to play a role in the aggressiveness of cancer. These microvesicles are more frequently released by tumor cells and may facilitate communication within the local microenvironment and the primary tumor (Baj-Krzyworzeka et al., 2006; Valadi et al., 2007; Huber et al., 2008; Iero et al., 2008). Patient-derived cancer-associated fibroblast exosomes have been shown to alter the cellular metabolism of prostate and pancreatic tumor cells *in vitro*, redirecting from oxidative phosphorylation to a glycolysis and glutamine-dependent reductive carboxylation (Zhao et al., 2016). This study indicates the impact exosomes released by cells within the tumor microenvironment have on the cellular function of the tumor cells. Communication between the tumor microenvironment and the cancer cells supports tumor cell dissemination and early events in metastasis (Hood et al., 2009, 2011). Exosomes may have the ability to promote metastasis via the horizontal transfer of proteins, miRNAs and other molecules to recipient cells (Ratajczak et al., 2006; Aliotta et al., 2010; Balaj et al., 2011; Peinado et al., 2012). Exosomes containing the RNA-binding protein LIN28 (which is a known marker of poor outcome for ovarian cancer) were shown to be taken up by recipient cells and significantly increase transcription of genes involved in Epithelial-to-Mesenchymal Transition (EMT), cell migration and invasion in the recipient cells (Enriquez et al., 2015).

## Metastasis

Metastasis is the major cause of cancer-related death (Mehlen and Puisieux, 2006) that occurs in a stepwise fashion relying on a number of host-tumor interactions (Fidler and Hart, 1982; Pauli et al., 1983). In order for a metastatic tumor to form, a cell from the primary tumor must have the ability to survive on its own, dissociate from the tumor, occupy the surrounding tissue (Liotta and Stetler-Stevenson, 1991), enter circulation, survive the environment of the circulatory system, invade the distant parenchyma and proliferate on its own (Liotta and Stetler-Stevenson, 1991). Circulating tumor cells (CTCs) can be found in the vasculature of various organs but only in some organs where a secondary tumor will survive and develop into sites of metastasis (Poste and Fidler, 1980). It has been noted that primary tumors preferentially home to particular organs. For example, melanoma preferentially metastasizes to the lung and brain (Fidler, 2003), therefore, successful metastatic growth is dependent on a microenvironment that is receptive of that particular cancer cell type (Fidler, 2003). Aberrant expression of GPCR proteins has been suggested to play a role in the organ-specific metastasis of cancer cells by way of enhancing mobilization, promoting angiogenesis and proliferation (Lee et al., 2008). To develop therapies focused on treating metastatic diseases, understanding the molecular mechanisms of metastasis is vital. Although the disseminated primary tumor cells are essential to metastasis, the cells from the surrounding tumor microenvironment are equally critical in prompting metastatic ability.

## Formation of the pre-metastatic niche

The formation of the pre-metastatic niche is an essential step in successful metastatic growth. The primary tumor initiates this formation by releasing factors into circulation and exosomes released from the tumor have been implicated in this process. Peinedo et al., described the involvement of exosomes in tumor progression and in the preparation of the pre-metastatic niche of future secondary tumor sites in a melanoma model system (Peinado et al., 2012). They provided evidence that exosomes are released by the primary tumor into the circulation, which results in the leakiness of the vasculature, as well as recruitment of immune cells, both events are involved in pre-metastatic niche formation (Peinado et al., 2012).

## Changes within the pre-metastatic environment

Exosomes released by tumor cells contain factors such as macrophage migration inhibitory factor (MIF) that influence the physiology of the recipient cells. The engulfment of MIF-containing exosomes promotes the release of transforming growth factor beta (TGFβ) by Kupffer cells, which then initiates the production of fibronectin by the hepatic stellate cells (hStCs; Costa-Silva et al., 2015). Resident fibroblasts and cells from the primary tumor stimulate fibronectin deposition (Kaplan et al., 2005; Erler et al., 2009). The deposition of fibronectin within the organs determines the location of the metastatic niche formation (Kaplan et al., 2005). Fibronectin deposited within the tissue causes the arrest of bone marrow derived cells (BMDC), specifically macrophages and neutrophils, within the deposits (Erler et al., 2009).

In addition to fibronectin, fibroblasts express Tenascin-C (TN-C) glycoprotein, within the premetastatic site, which may protect the cancer cells from apoptosis (O'Connell et al., 2011). Several cytokines, as well as Wnt and Ras/MAPK signaling, could induce TN-C glycoprotein expression. TN-C is not found in normal tissues, however, under pathological conditions, such as inflammation and cancer, its protein expression is strikingly increased and induces the production of angiogenic protein factors such as MMP-9. TN-C also has been implicated to affect steps in cancer progression including proliferation, migration, invasion and angiogenesis. Reviewed by Tse and Kalluri (2007).

Periostin is a secretory protein also deposited within the extracellular matrix (ECM) by fibroblasts, which acts as a bridge that binds to TN-C as well as fibronectin and collagen (Kii et al., 2010; Wang and Ouyang, 2012). Studies showed that periostin did not have a direct effect on the growth of tumor cells, however, knocking out periostin leads to a significant reduction in the metastatic potential (Wang and Ouyang, 2012). Versican is an extracellular matrix (ECM) proteoglycan that is expressed by myeloid cells present in the pre-metastatic niche. It is involved in mesenchymal to epithelial transition by decreasing phospho-Smad2 levels, which increases proliferation and metastasis, but does not play a role in the recruitment of immune cells or the manipulation of the immune environment (Gao et al., 2012).

In addition to remodeling the extracellular matrix to create greater permeability within the surrounding vasculature, which is necessary in forming a pre-metastatic niche that is receptive of CTCs, the vasculature is manipulated as well. Vascular remodeling occurs to allow for the extravasation of CTCs out of circulation, into the pre-metastatic environment. This process is dependent on angiopoietin 2 (Angpt2), matrix metalloproteinase 3 (MMP-3), and MMP-10. Huang et al., showed that knocking down these proteins reduces the vascular permeability and decreases the infiltration of myeloid cells and inhibits spontaneous lung metastasis in an *in-vivo* model (Huang et al., 2009).

In a breast cancer exosome model, the macrophages within the lung and brain both phagocytose exosomes, which results in the activation of NF-kB and subsequent release of pro-inflammatory cytokines such as IL-6, TNFα, GCSF, and CCL2, which promote metastasis development (Chow et al., 2014). Hypoxic breast cancer cells release an amine oxidase, lysyl oxidase (LOX) that accumulates at sites of pre-metastatic niche formation. LOX co-localizes with metastases and crosslinks collagen within the basement membrane and is essential for the recruitment and adherence of myeloid cells. This crosslinking is critical for CD11b^+^ myeloid cell recruitment, which led to interactions with the collagen and production of MMP-2, breaking down collagen into peptides that act as chemoattractants for bone marrow derived cells (BMDCs) and circulating tumor cells (CTCs; Erler et al., 2009).

## Recruitment of immune cells

Exosomes have the ability to “educate” bone progenitor cells to be receptive of and support tumor cell growth and metastasis (Peinado et al., 2012). BMDCs express vascular endothelial growth receptor 1 (VEGFR1), which may be responsible for the homing of tumor cells to the pre-metastatic niche. Erler et al., showed accumulation of VEGFR1^+^ BMDCs in common sites of metastasis in the lung, within 9 days post-accumulation, micrometastases formed and BMDCs remained within the site (Erler et al., 2009). As described earlier, fibronectin deposition within the pre-metastatic environment will result in the arrest of bone marrow derived cells. When the BMDCs arrive, they form clusters of cells in the tissue parenchyma at common sites of metastasis before evidence of tumor cells (Kaplan et al., 2006). VEGFR1^+^ hematopoietic cells (HPCs) express VLA-4, which allows them to adhere to the newly synthesized fibronectin to initiate the cellular clustering (Kaplan et al., 2006). Interaction of VLA-4 with fibronectin is responsible for the ability of HPCs to move within the bone marrow (Burger et al., 2003). After fibronectin binding in HPCs, MMP protein expression is enhanced with the presence of integrin signaling (Huhtala et al., 1995; Yakubenko et al., 2000). MMP-9 functions to breakdown basement membranes and the release of Kit-ligand and VEGF-A, presumably to support bone marrow migrating cells that express c-Kit (Bergers et al., 2000; Heissig et al., 2002).

Myeloid cell recruitment is influenced by the protein expression of several inflammatory chemoattractants, which are influenced by the primary tumor. These chemoattractants recruit Mac1^+^ (macrophage antigen 1) myeloid cells to the lung. Furthermore, Hiratsuka et al., found these chemoattractants were involved in the ability of the tumor cells to migrate, using pseudopodia for invasion. When the protein expression of these inflammatory chemoattractants was abolished, migration of both tumor cells and Mac1^+^ myeloid cells was prevented (Hiratsuka et al., 2006).

## GPCRs and exosomes

A potential relationship between GPCRs and MVB formation, exosome endocytosis, or exosome release has been suggested. For example, the G protein-coupled pheromone receptor, Ste2, is downregulated after activation by the transfer of the receptor to the lumen of the vacuole by way of MVB sorting (Odorizzi et al., 1998). However, Myers et al., showed that activation of GPCRs result in growth factor shedding by way of proteolytic cleavage, and not by exosome release (Myers et al., 2009). Therefore, certain GPCRs, but not all, may play a role in the MVB exocytosis. Some GPCRs, specifically A_2*A*_ receptors, have been shown to have the ability to be transferred by exosomes from a source cell expressing these receptors to a target cell that does not. Upon incubation with an A2A receptor agonist, the target cells produced an increased amount of cAMP, suggesting that the transferred receptor was then shown to be functionally active within the target cell (Guescini et al., 2012). Additionally, under cellular stress responses to neurohormonal stimulation, cardiomyocytes are stimulated to release exosomes containing an endogenous functional GPCR, AT1R, which, upon activation with an AT1R agonist, results in phosphorylated-ERK (Pironti et al., 2015). These studies suggest that functioning GPCRs can be transferred by exosomes, influencing physiological changes within the recipient cell. Locke et al., identified the relationship between the activation of GPR143 by its natural ligand, L-DOPA, in retinal pigment epithelial cells, and the release of exosomes for intercellular communication in the eye (Locke et al., 2014). Downstream exosome release is dependent on the interaction of L-DOPA with the receptor, which activates Gα_q_, initiating the release of calcium storage from the cell. Calcium mobilization has been suggested to play a role in the release of exosomes (Savina et al., 2003; Pant et al., 2012).

**Figure 1:**
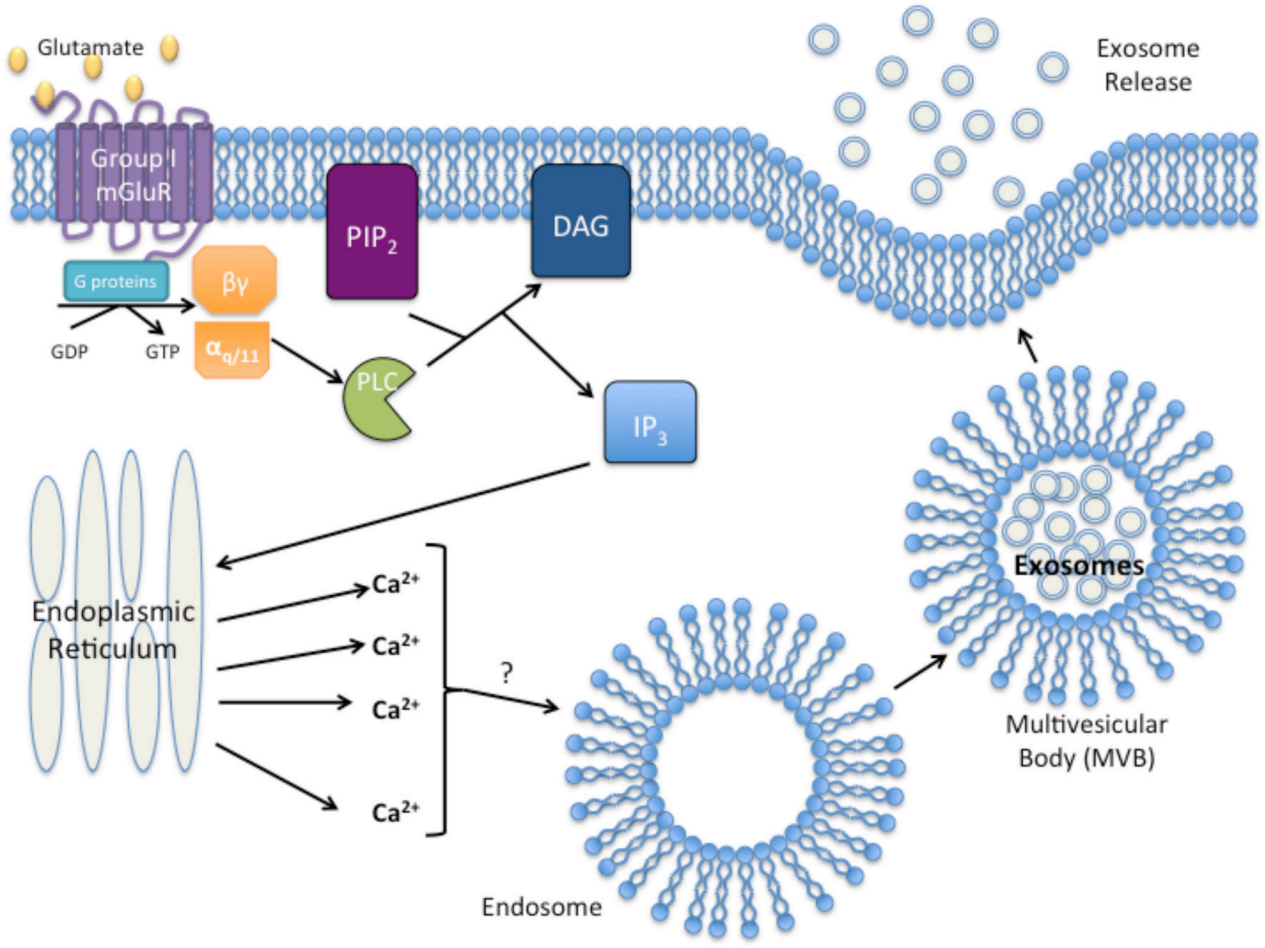
**Proposed model of group I Metabotropic glutamate receptor (mGluR) activation and exosome release**. Activation of group I mGluR by glutamate results in the intracellular G-protein exchange of guanine diphosphate (GDP with guanine triphosphate (GTP). Exchange results in the activation of the α_*q*∕11_ subunit and activation of phospholipase C (PLC). PLC then cleaves phosphatidylinositol 4,5-bisphosphate (PIP_2_) into diacylgycerol (DAG) and inositol triphosphate (IP_3_). IP_3_ initiates release of Ca^2+^ from the endoplasmic reticulum. Excess intracellular Ca^2+^ initiates exosome formation/release through an unknown mechanism.

Given the examples of GPCR activation resulting in exosome formation, release, and uptake, it seems logical to suggest a potential role of GPCRs in exosome biogenesis and function. Furthermore, activated group I mGluRs promote the release of calcium from the endoplasmic reticulum by the second messenger, IP_3_, and increased intracellular calcium levels have been suggested to result in the release of exosomes (Savina et al., 2003; Pant et al., 2012). Interestingly, activated phospholipase C (PLC) that hydrolyzes PIP_2_ for IP_3_ formation was detected within exosomes of a leukemia cell line, suggesting that exosomes may carry functional phospholipases to recipient cells (Subra et al., 2010). Modulation of calcium concentration may be a potential link between group I mGluR activation and exosome release as depicted in Figure 1. This association between mGluRs and exosome release may provide hints to elucidate the aggressive nature of cancers that ectopically express mGluRs, and the role exosomes play in the metastatic potential of the tumor, and formation of the pre-metastatic niche.

## Conclusions

Taken together, the aggressiveness and malignancy exhibited by cancers aberrantly expressing GPCRs could be explained by the release of a high volume of exosomes not only manipulating the surrounding stromal of the tumor, but also preparing the sites of future metastasis for the arrival of a circulating tumor cell. We hypothesize that stimulation of GPCR by it ligand/agonist initiates signaling cascades, activating a multitude of different downstream effectors that may regulate exosomal secretion and/or production. The precise mechanisms remain unknown. Calcium has been proposed as one of the “factors” involved, for example, stimulated group I mGluRs activate PLC and promote hydrolytic cleavage of PIP_2_ for the formation of two second messengers, IP_3_ and DAG. IP_3_ brings about the release of calcium from the endoplasmic reticulum, which initiates multiple diverse physiological alterations within the cell; one of them could be exosome release. Therefore, it is plausible that GPCR signaling may participate in exosome production or secretion by tumor cells.

## Author contributions

This review was written by AI, who works under the guidance of SC, the principle investigator.

## Funding

This review was funded by the National Institute of Health (R21CA185835) and The Bristol-Myers Squibb Graduate Research Fellowship in Toxicology.

### Conflict of interest statement

The authors declare that the research was conducted in the absence of any commercial or financial relationships that could be construed as a potential conflict of interest.
